# Variation in the expression of a transmembrane protein influences cell growth in *Arabidopsis thaliana* petals by altering auxin responses

**DOI:** 10.1186/s12870-020-02698-5

**Published:** 2020-10-22

**Authors:** Charlotte N. Miller, Jack Dumenil, Fu Hao Lu, Caroline Smith, Neil McKenzie, Volodymyr Chapman, Joshua Ball, Mathew Box, Michael Bevan

**Affiliations:** Cell and Developmental Biology Department John Innes Centre, Norwich Research Park, Norwich, NR4 7UH UK

**Keywords:** *Arabidopsis thaliana*/organ size variation/natural genetic variation/auxin responses

## Abstract

**Background:**

The same species of plant can exhibit very diverse sizes and shapes of organs that are genetically determined. Characterising genetic variation underlying this morphological diversity is an important objective in evolutionary studies and it also helps identify the functions of genes influencing plant growth and development. Extensive screens of mutagenised Arabidopsis populations have identified multiple genes and mechanisms affecting organ size and shape, but relatively few studies have exploited the rich diversity of natural populations to identify genes involved in growth control.

**Results:**

We screened a relatively well characterised collection of *Arabidopsis thaliana* accessions for variation in petal size. Association analyses identified sequence and gene expression variation on chromosome 4 that made a substantial contribution to differences in petal area. Variation in the expression of a previously uncharacterised gene At4g16850 (named as *KSK*) had a substantial role on variation in organ size by influencing cell size. Over-expression of *KSK* led to larger petals with larger cells and promoted the formation of stamenoid features. The expression of auxin-responsive genes known to limit cell growth was reduced in response to *KSK* over-expression. ANT expression was also reduced in *KSK* over-expression lines, consistent with altered floral identities. Auxin responses were reduced in *KSK* over-expressing cells, consistent with changes in auxin-responsive gene expression. *KSK* may therefore influence auxin responses during petal development.

**Conclusions:**

Understanding how genetic variation influences plant growth is important for both evolutionary and mechanistic studies. We used natural populations of *Arabidopsis thaliana* to identify sequence variation in a promoter region of Arabidopsis accessions that mediated differences in the expression of a previously uncharacterised membrane protein. This variation contributed to altered auxin responses and cell size during petal growth.

**Supplementary information:**

The online version contains supplementary material available at 10.1186/s12870-020-02698-5.

## Background

Cell proliferation and cell growth are coordinated to generate the characteristic sizes, shapes and functions of plant organs. This coordination involves multiple cellular processes, including signaling mechanisms, cell division, turgor-driven cell expansion, and cell wall and protein synthesis [1]. During the formation of determinate plant organs such as leaves and petals, cell proliferation with limited cell growth occurs at earlier stages of organ formation, followed by cell growth with limited cell proliferation, which occurs to increase cell size, accompanied by differentiation as the developing organ attains its final characteristic size and shape [2]. Very little is known about the spatial and temporal integration of cell proliferation and cell growth to generate the final sizes and shapes of organs and seeds, despite its fundamental and applied importance.

Many plant species display a wide range of forms due to altered sizes and shapes of organs, reflecting adaptation to their natural environments. The natural range of the annual species *Arabidopsis thaliana* extends from northern Scandinavia to Africa, and it exhibits a correspondingly diverse range of phenotypes [3–5], such that most accessions are phenotypically distinct. However, genetic variation underlying this phenotypic variation is still poorly understood. For example, the extent to which variation in the functions of genes influencing organ size established in one experimental accession influences natural variation in organ sizes in populations of *Arabidopsis thaliana* is not well understood. Also, the extent of conservation of known mechanisms influencing organ size and many other traits in natural populations is also insufficiently documented. Therefore, an increased understanding of the genetic foundations of natural variation in traits such as organ size will shed light on how natural genetic variation influences mechanisms controlling organ size and other traits.

*Arabidopsis thaliana* has adapted to diverse habitats worldwide, with extensive natural variation in organ size reflecting these different life histories [4]. Although variation in the shapes and sizes of different floral organs are correlated in order to maintain the reproductive functions of the flower [6], significant genetic variation influencing several floral morphology traits was identified by QTL analyses of Arabidopsis Recombinant Inbred Line (RIL) populations [7, 8]. More recently, QTL analyses identified six independent loci influencing variation in petal shape and size, with variation at the *ERECTA (ER)* locus accounting for 51% of this variation [9]. Haplotype variation in 32 accessions at the *GA1* locus was associated with variation in petal, stamen and style lengths [10]. In one of the few studies exploiting natural variation to identify a previously unknown growth regulator, *BRX* was established as a regulator of cell proliferation during root growth [11]. Despite these studies, there are limited examples of the identification and characterisation of natural variation in organ size in Arabidopsis.

Genome-wide association (GWA) mapping in Arabidopsis is increasingly used to access a wider range of natural genetic variation, to identify small-effect alleles, and to map genotype-phenotype relationships more precisely [12]. The very small size of their genomes has facilitated the re-sequencing of a large range of *Arabidopsis thaliana* accessions and the identification of vast numbers of SNP and small indel variants by comparison to the assembled Col-0 accession [13]. Within this wide-ranging set of accessions, those from Sweden are relatively well documented [14] and have been screened for variation in over-wintering responses [15]. Initial inspection of this collection showed considerable variation in petal size and shape, therefore we conducted an association analysis of 272 Swedish accessions. We identified variation in the promoter of a hypothetical gene At4G16850, predicted to encode a 6-transmembrane (6TM) protein. Accessions with increased At4g16850 expression had larger petals due to increased cell growth. This was confirmed by transgenic lines over-expressing the coding region of At4g16850, which was called *KSK*. Over-expression of At4g16850 lowered expression of several auxin-responsive genes that modulate petal cell size and also reduced auxin responses. At4g16850 over-expression also led to the partial homeotic conversion of petals to stamenoid structures, and this was attributed to altered expression of floral organ development genes.

## Results

### Identifying a locus influencing petal area

**Fig. 1:**
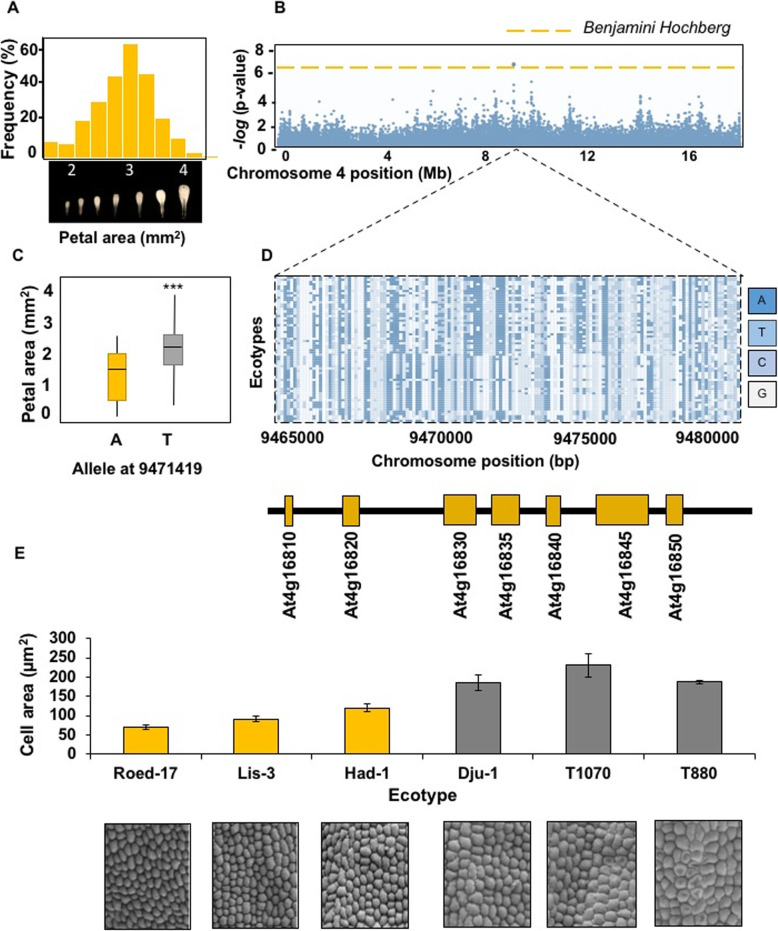
Association of petal area variation with sequence variation in Swedish accessions of *Arabidopsis thaliana*. **a** Normal distribution of the petal area trait in the set of 272 accessions collected from Sweden (Additional File 1). **b** Associations between 250,000 SNPs and petal areas determined by GWAPP using an Accelerated Mixed Model. The dotted line represents significance determined by the Benjamini-Hochberg FDR. Positions on chromosome 4 are shown in Mb, and associations as -log10 *P* values. **c** Petal areas in Arabidopsis accessions with the A or T allele at position 9,471,419 bp on chromosome 4. *** represents *P* < 0.001. **d** Linkage block of haplotypes associated with large petal areas. Haplotypes extending across 15 kb containing 6 gene models can be seen. The location and identity of gene models is shown in the lower panel. **e** Petal abaxial cell areas of three Swedish accessions carrying the increasing T allele (gray bars) and three containing the reducing A allele (orange bars)

We measured the length, maximum width and area of petals of 272 *Arabidopsis thaliana* accessions collected from southern and northern Sweden (Additional File 1) that were grown in controlled conditions after vernalization. Additional File 2 shows petal phenotype measurements. All three petal parameters varied substantially within the sampled collection. For example, mean petal areas varied from 0.915 mm^2^ (Hov1–10) to 4.92 mm^2^ (Vår2–6), a difference of 537%. Additional File 3 shows representative petals from these accessions and from Död 1, an intermediate size for comparison. Fig. 1a shows that mean petal area variation formed a normal distribution and was therefore suitable for association studies. GWAPP was used [16] with an Accelerated Mixed Model, incorporating information across 250,000 SNPs. This analysis identified a significant SNP association on chromosome 4 for petal area (Fig. 1b). The most significantly associated SNP within this region was located at position 9,471,419 bp, within gene model At4G16830. The marker at this position was bi-allelic, with those accessions carrying a “T” allele at this locus exhibiting a ~ 15% increase in petal area relative to those carrying the alternative “A” allele (Fig. 1c). The extent of Linkage Disequilibrium (LD) in the region was visualised using information for all SNP markers within +/− 10 kb the 9,471,419 bp position from each accession and colour-coded based on the allele present. These markers, in chromosome order, were then sorted by phenotype values. This identified a clear block of LD (Fig. 1d), with accessions exhibiting larger petals carrying a distinctive set of alleles from those with smaller petals. This block of LD spanned six Arabidopsis gene models, from At4g16820 to At4g16850. Sequence variation altering the activities of any of these genes may explain the variation in petal size observed across accessions. Assessment of gene annotations revealed no known regulators of petal or organ size. The effect of genetic variation within the haplotype defined by LD on petal growth was assessed in a subset of three accessions with small petal areas and three accessions with large petal areas (Fig. 1e). Petal cell areas and numbers were quantified using Scanning Electron Microscopy (SEM) and Image J. A significant increase in petal abaxial epidermal cell area was observed in accessions carrying the increasing T allele at position 9,471,419 relative to accessions carrying the decreasing A allele (Fig. 1e). Therefore, the major effect of genetic variation in the haplotype was on petal cell area.

### Expression levels of At4g16850 are correlated with quantitative variation in petal size

**Fig. 2:**
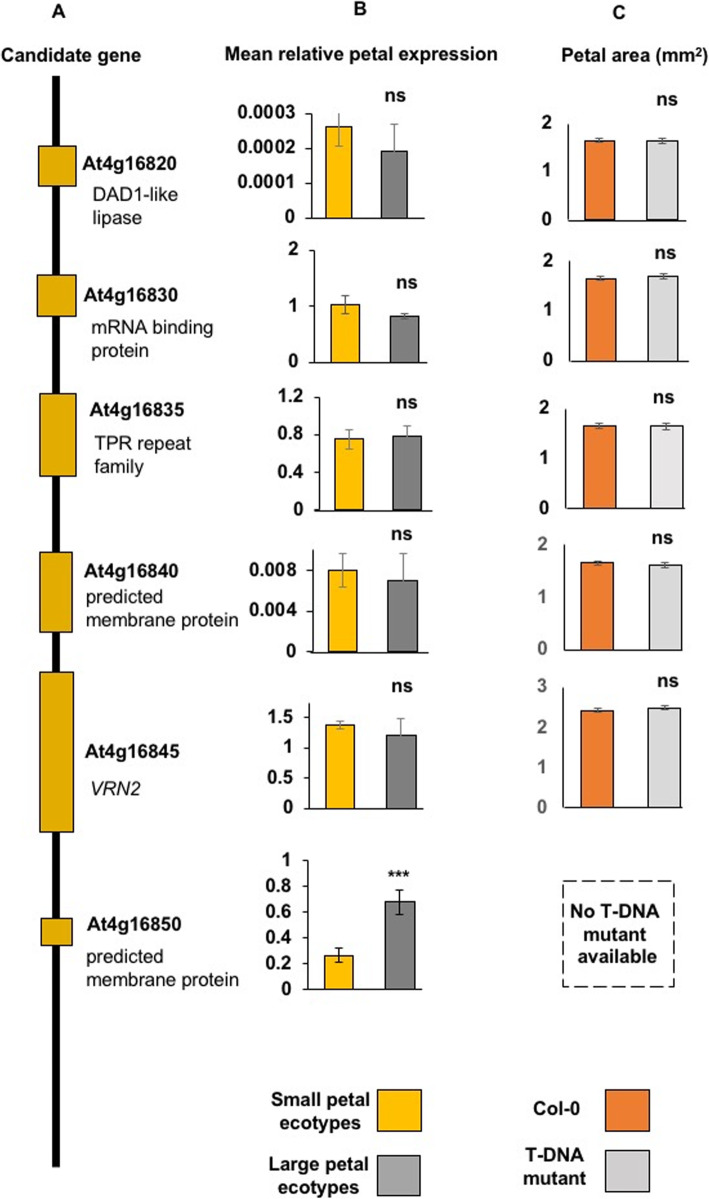
Expression levels and petal area phenotypes of candidate genes. **a** Representation of predicted genes in the 15 kb region of LD associated with variation for petal size. Predicted and known functions of genes in the LD block are shown. The diagram is not to scale. **b** Mean gene expression levels of candidate genes in petals of three small and three large petal accessions. These are shown in Fig. 1e. Expression of At4g16850 was significantly increased (*P* < 0.001) in large petal accessions. Expression levels are relative to *EF1ALPHA* gene expression. Data are given as means of +/−SE (*n* = 3 biological replicates. *P* values were determined by Student’s *t*-test. ns is not significant. **c** Petal areas of T-DNA insertion alleles of candidate genes in accession Col-0. ns is not significant. No T-DNA mutant was available for At4g16850

To assess the potential role of the 6 candidate genes in regulating petal growth, petal areas were measured in available potential loss-of-function T-DNA mutants in the accession Col-0. T-DNA insertion lines were available from stock centres for all genes found to be in high LD with associated markers with the exception of At4g16850, a small hypothetical gene of unknown function. We measured the expression of these 6 candidate genes in developing floral tissues in the six accessions with varying petal sizes. For At4g16820 to At4g16845 no differences in petal area were seen in the T-DNA insertion lines relative to Col-0 plants (Fig. 2c). Furthermore, no differential expression of these genes in developing flowers was observed between the six accessions with small and large petals (Fig. 2b). However, for At4g16850, a gene of unknown function, there was an increase in petal transcript levels in the Dju-1, T1070 and T880 accessions with larger petals (*P* ≤ 0.001) (Fig. 2b). At4g1650 expression was also increased in seedlings of these larger petal accessions (Additional File 4) compared to the Col-0 accession.

### Polymorphisms in the At4g16850 gene region

**Fig. 3:**
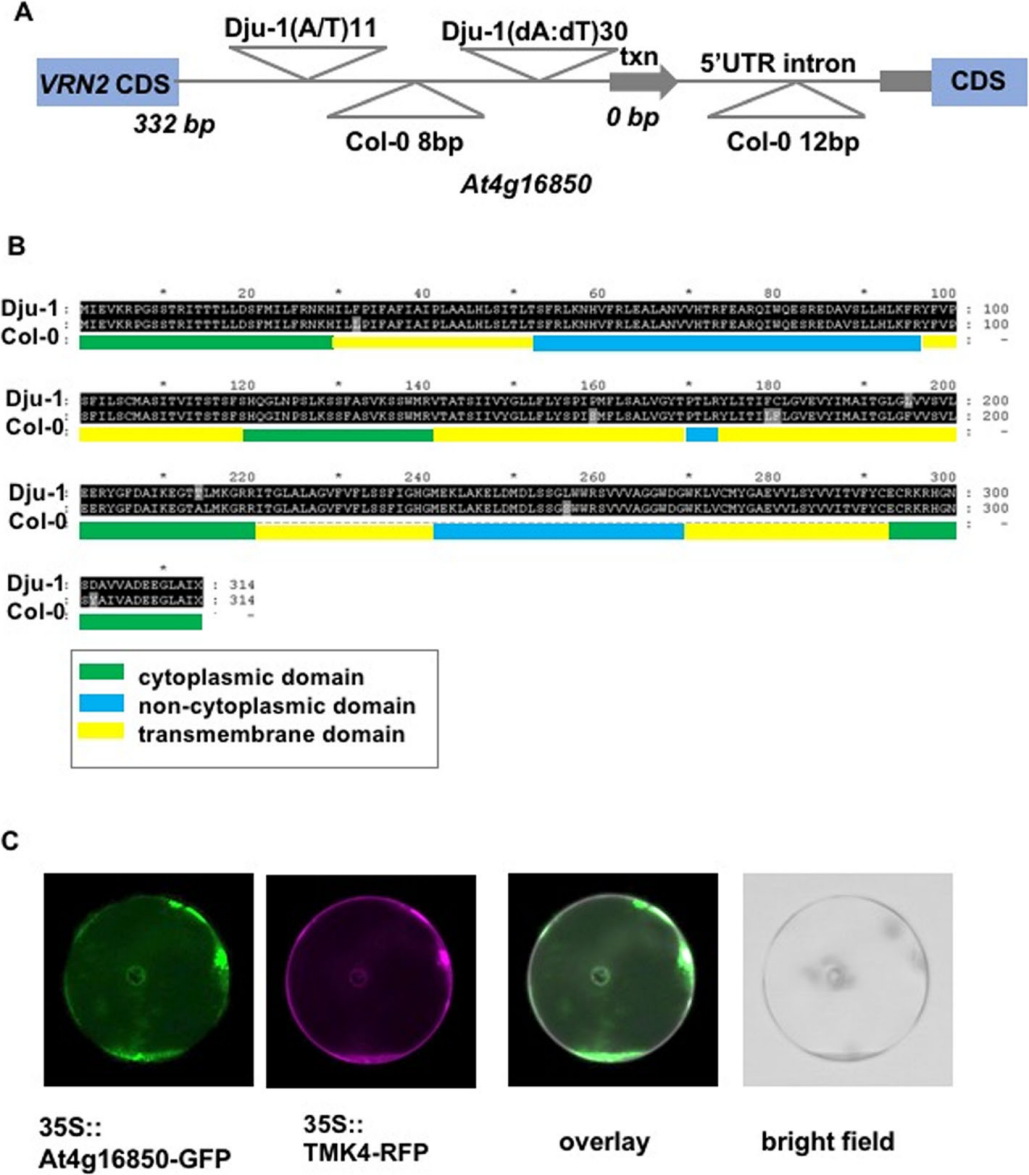
Characterisation of candidate gene At4g16850. **a** Sequence variation in the 322 bp intergenic and 5’UTR region between VRN2 and At4g16850 transcription start site, and in the 5’UTR, between the large petal accession Dju-1 and small petal genotype Col-0. Two A/T insertions in Dju-1 relative to Col-0 are shown above the line, and insertions in Col-0 relative to Dju-1 are shown below the line. Additional File 5 shows a sequence alignment of these regions. **b** The coding regions of At4g16850 from the large petal accession Dju-1 and the small petal accession Col-0 were aligned to identify predicted protein sequence differences. The coding regions were analysed with InterPro to identify putative transmembrane and cytoplasmic protein domains, shown as coloured bars under the predicted coding sequence. Amino acid differences are shown as gray highlights. **c** Transient expression of At4g16850 coding region fused to GFP at its C-terminus from the 35S promoter in Col-0 petal protoplasts. A known plasma-membrane protein TMK4 fused to RFP was used to reveal co-location in the plasma membrane. The white colour in the overlay reveals co-location of At4g16850-GFP and TMK4-RFP

The relationship between increased petal areas and increased expression of At4g16850 in the selected accessions suggested that sequence variation between accessions may influence At4g16850 expression. Inspection of available genome sequence reads [13] from the larger petal accessions Dju-1, T1070 and T880 showed a limited range of SNP and possible small indel variation in the coding region and flanking sequences compared to the Col-0 assembly. To identify a wider range of promoter sequence variation, a region 2 kb upstream of At4g16850 in three accessions with the decreasing A allele (Roed-17, Lis-3, Had-1) and three with the increasing T allele (Dju-1, T1070, T880) were amplified by PCR, cloned and sequenced to identify the precise location and types of sequence variation in the putative promoter regions of At4g16850. Primers were designed in conserved regions of all accessions. The upstream regions were readily amplified from the three smaller petal accessions and were found to be very similar to the sequence of the Col-0 promoter in accessions carrying the decreasing A allele (Fig. 1c), consistent with its relatively small petal phenotype. Col-0 was therefore selected as the “small petal” reference genome due to the high level of sequence conservation between small petal accessions and Col-0 at the locus. However, no full-length promoter amplicon could be generated from any of the large petal accessions. We therefore generated whole genome assemblies from Illumina sequence of un-amplified DNA templates [17] made from three large petal accessions to access sequence variation in At4g16850. An ABYSS de novo assembly generated a large contig spanning the region upstream of the At4g16850 in line Dju-1. Comparison to the Col-0 small petal sequence identified multiple variants (Fig. 3a and Additional File 5). Notably, the Col-0 and Dju-1 promoters had a common 23 bp dA:dT-rich region that was extended by 30 bp in the Dju-1 promoter, making an approximately 50 bp dA:dT-rich region in Dju-1. It is likely that this dA:dT richness impeded PCR amplification of full-length upstream regions of large-petal accessions. There were also many other promoter polymorphisms, including another large dA/T-rich insertion in the intergenic region of Dju-1 compared to Col-0, and a deletion in Dju-1 compared to Col-0 in the 5’UTR intron (Fig. 3a).

At4g16850 encodes a predicted 6-transmembrane domain protein with 3 non-cytoplasmic domains and 4 cytoplasmic domains (Fig. 3b). Comparison of the Dju-1 and Col-0 assemblies revealed the predicted protein was highly conserved between these large- and small-petal accessions, with only two non-conservative amino acid changes in trans- membrane region 4 and in the C-terminal cytoplasmic domain (Fig. 3b). To assess the predicted subcellular location of the protein encoded by At4g15850, its coding region was fused at its C-terminus with GFP and transiently expressed from the 35S promoter in Col-0 developing petal protoplasts, together with a known transmembrane receptor-like kinase TMK4 [18] fused to RFP. Confocal imaging showed that the At4g16850-GFP fusion protein co-localised with the RFP-tagged TMK4 plasma membrane protein (Fig. 3c), demonstrating that it can be localised to the plasma membrane. At4g16850-GFP fusion protein was also observed in cytoplasmic structures.

### Overexpression of At4G16850 increases petal size due to increased cell growth

**Fig. 4:**
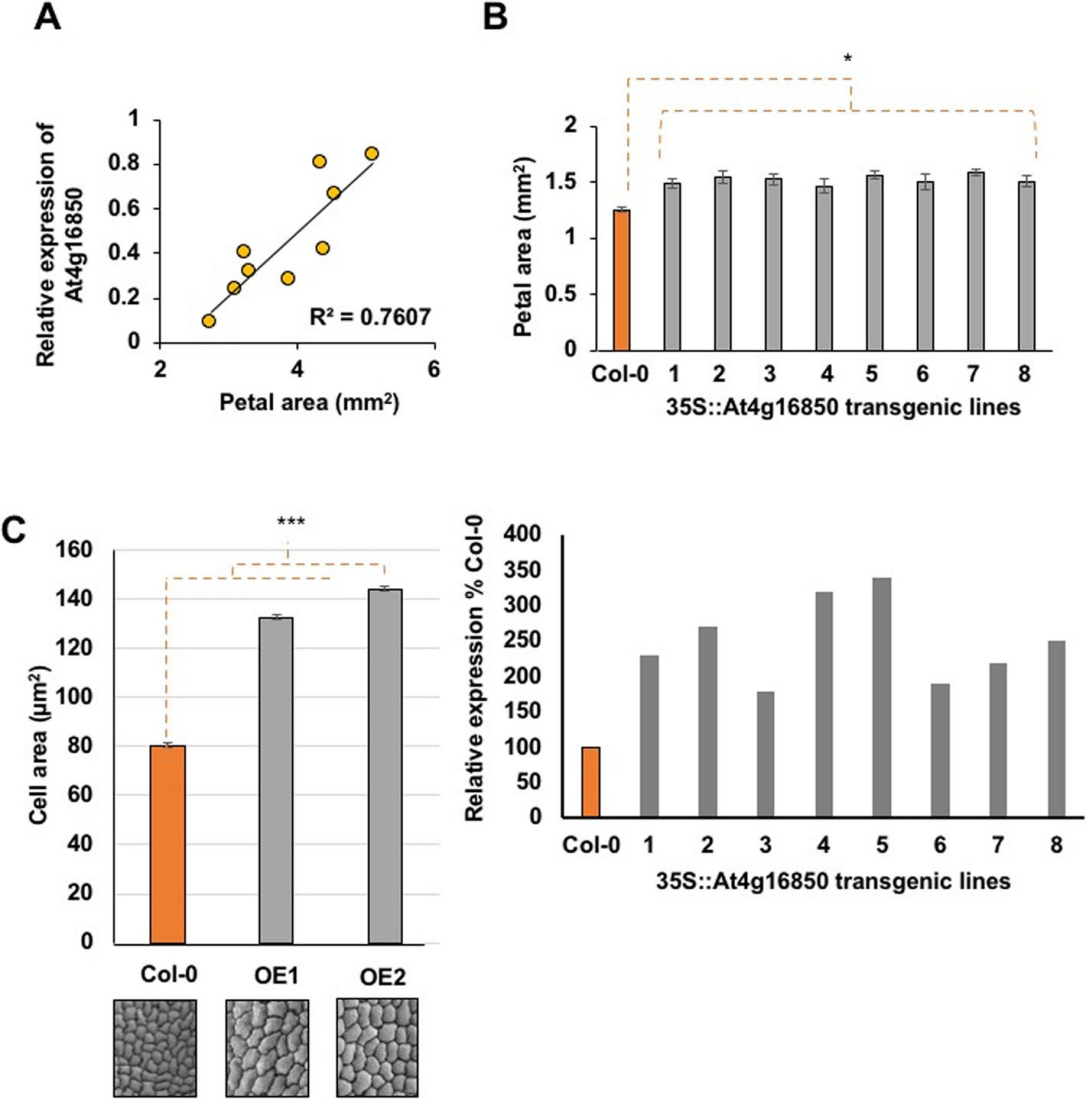
Over-expression of At4g16850 leads to larger petal cells. **a** Correlation of At4g16850 gene expression and petal areas in accessions exhibiting a wide range of petal areas. **b** Petal areas in transgenic Col-0 lines overexpressing the At4g16850 coding region from the 35S promoter. Eight independent transgenic lines with elevated At4g16850 transcript levels in petals were selected and petal areas measured (*n* = 50 petals, *P < 0.05). The lower panel shows relative expression levels of At4616850 in the transgenic lines compared to Col-0. **c** Cell areas on the abaxial side of petals from two selected over-expressing lines (OE1 and OE2) described in panel B above are increased (***P < 0.001, *n* = 50 cells)

Analysis of the expression of At4G16850 across accessions displaying high variation for petal area established that differential expression could account for 76% of the variation in petal size in the tested accessions (Fig. 4a). To establish whether this variation in At4g16850 caused petal size variation, the coding region of At4g16850 from Col-0 was expressed from the constitutive 35S promoter in transgenic Arabidopsis Col-0 plants. Col-0 has relatively small petals and inherits the decreasing allele in the associated haplotype that segregates with low At4G16850 expression. Therefore Col-0 was an appropriate accession in which to observe any expected increase in petal size following overexpression of At4g16850. Comparison of petal areas in transgenic lines and untransformed Col-0 plants revealed that all transgenic plants overexpressing At4G16850 (lower panel) exhibited significantly increased petal size relative to Col-0 (*P* ≤ 0.01) (Fig. 4b). Therefore, increased expression of At4g16850 leads to increases in petal area by approximately 125%, indicating that variation in At4g16850 expression among the accessions directly influences petal area. In the tested accessions exhibiting increased At4g16850 expression, cell areas were increased (Fig. 1e). Petal cell areas were also increased in transgenic lines overexpressing At4g16850 by approximately 175% (Fig. 4c). This suggested there were fewer larger cells in larger petals. Taken together, these results show that increased expression of At4g16850 promotes cell growth in Arabidopsis petals. To take account of this information about a previously unknown gene in *Arabidopsis thaliana* we named the gene *KSK* (KronbladStorleK, Swedish for petal size).

### Increased expression of *KSK* reduces expression of genes that limit petal cell growth

**Fig. 5:**
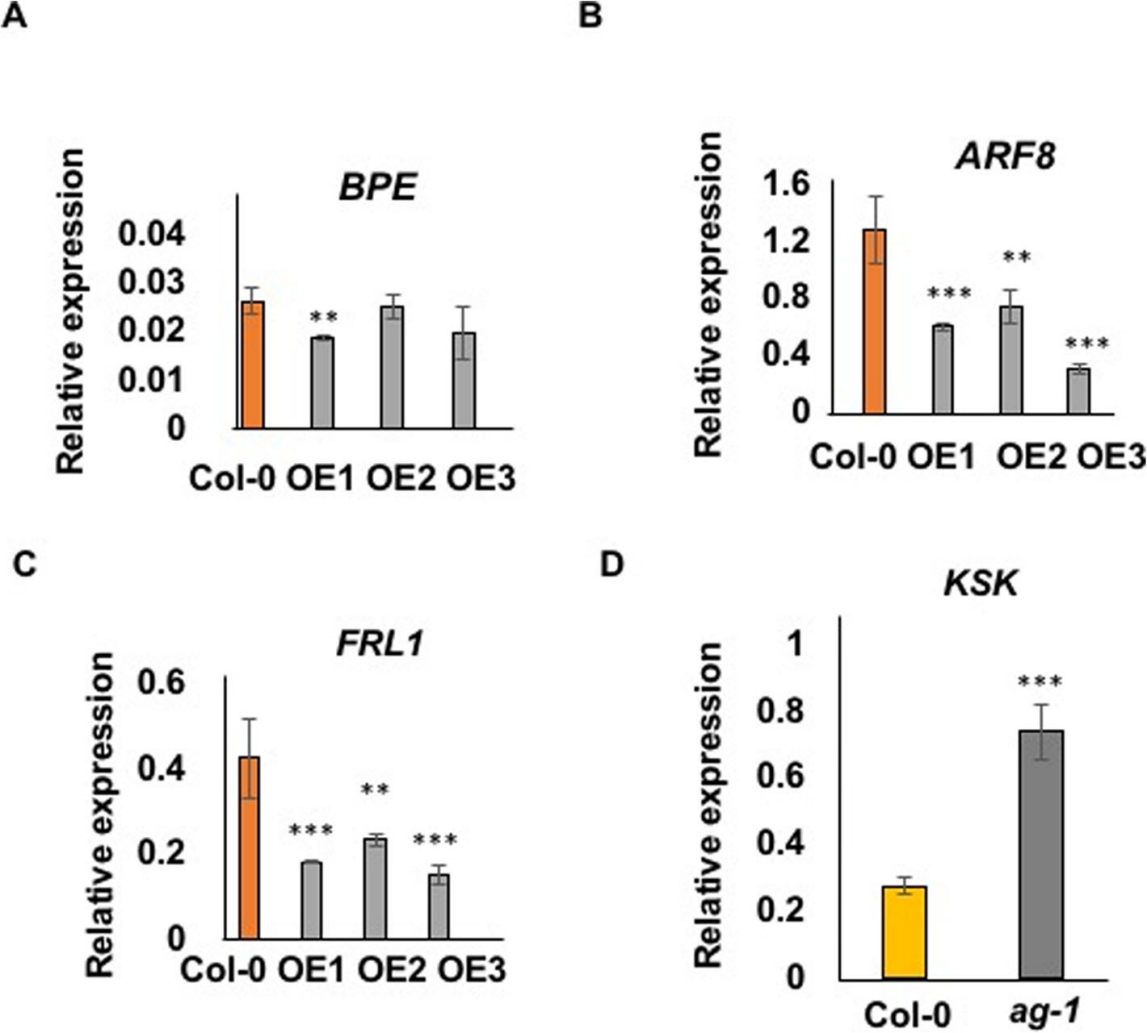
Reduced expression of petal cell growth genes in *KSK* over-expressing lines. Expression levels of *BPE* (**a**), *ARF8* (**b**) and *FRL1* (**c**) in developing petals of *35SS::KSK* overexpressing lines (OE 1,2,3). ** *P* < 0.01; *** *P* < 0.001. Expression levels are relative to *EF1ALPHA* gene expression. Data are given as means of +/−SE (*n* = 3 biological replicates. *P* values were determined by Student’s *t*-test. D. Expression of *KSK* in developing petals of Col-0 and the *agamous-1* mutant. Expression levels are relative to *EF1ALPHA* gene expression. Data are given as means of +/−SE (*n* = 3 biological replicates. *P* values were determined by Student’s *t*-test

Previous studies have identified several genes that influence petal cell growth in Arabidopsis. *BPEp* [19] and *ARF8* function together [20] to limit petal cell growth, and *FRL1* [21] also represses petal cell growth. The expression of these genes in developing petals of three transgenic Col-0 lines over-expressing At4g16850 and in untransformed Col-0 was measured using Q-RT-PCR to assess whether *KSK* may influence petal cell growth through these genes. Although only one transgenic line showed significant reduction in *BPE* expression in petals (Fig. 5a), consistent reductions in *ARF8* and *FRL1* expression in developing transgenic petals was seen (Figs. 5b, c) compared to Col-0. This suggested that *KSK* may promote petal cell growth by reducing the expression of these petal cell growth genes. *AGAMOUS* reduces BPEp expression [19] and the *ag-1* loss of function mutant has larger petals [22], consistent with the larger cell and petal sizes in BPEp loss of function mutants. Although we did not observe consistent reduction of *BPEp* expression in all *KSK* overexpressing transgenic lines, we tested whether *AG* influences *KSK* expression. *KSK* expression was doubled in the *ag-1* loss-of-function mutant (Fig. 5d) consistent with a model in which *AG* repressed *KSK* expression, leading to increased *ARF8* and *FRL1* expression and corresponding reduced petal cell size and overall petal area.

### Overexpression of *KSK* leads to partial homeotic conversion of petals to stamenoid structures

**Fig. 6:**
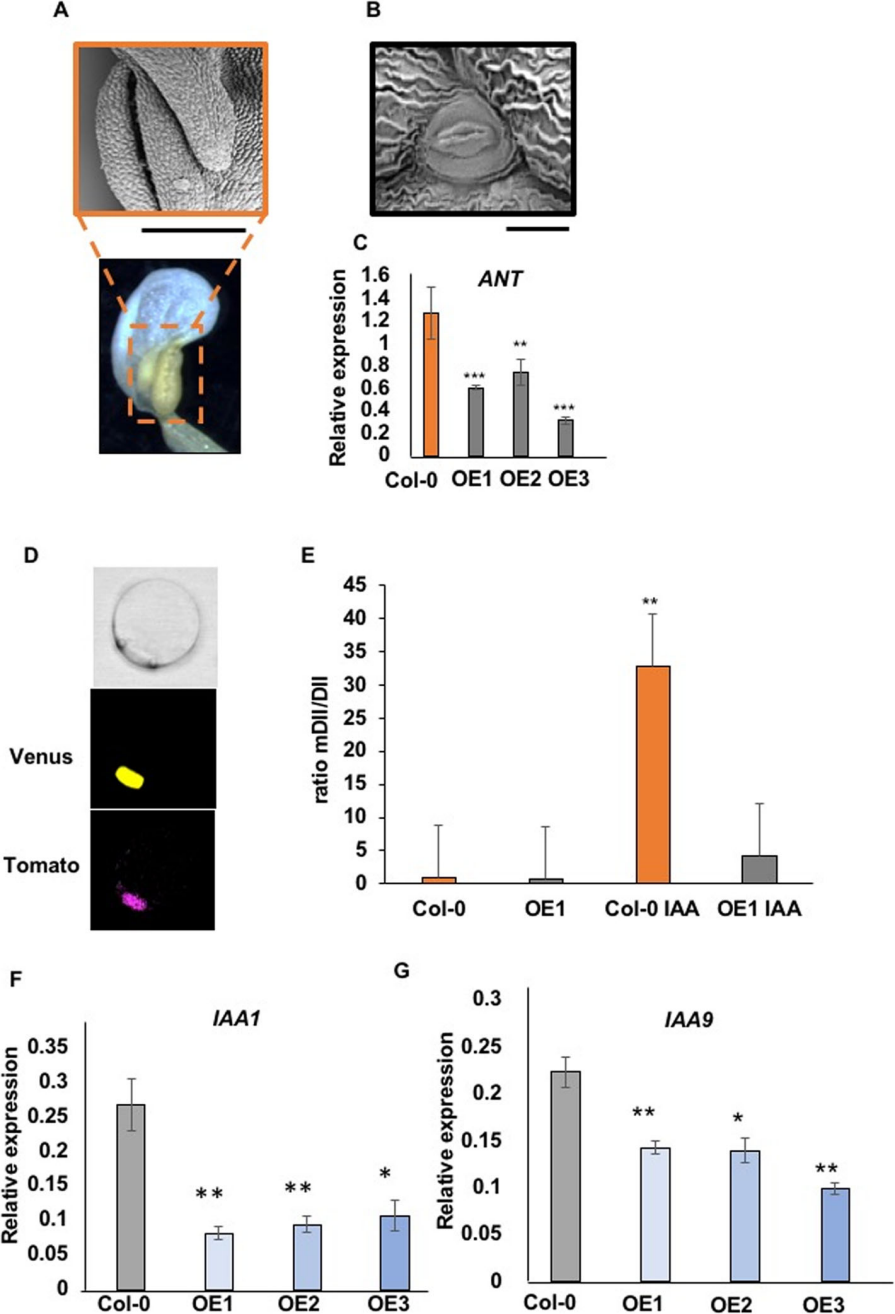
Over-expression of *KSK* leads to partial homeotic conversion of petals to stamenoid structures and leads to reduced auxin responses. **a** SEM (upper) and bright field (lower) image of a petal from a *35S::KSK* transgenic line showing stamenoid features such as a pollen sac. The scale bar represents 100 μm. **b** SEM of petal epidermal cells from a *35S::KSK* transgenic line showing a stomata. The scale bar represents 10 μm. **c** Relative expression of *ANT* in Col-0 and *35S::KSK* OE1, OE2 and OE3 developing petals. Expression levels are relative to *EF1ALPHA* gene expression. Data are given as means of +/−SE (*n* = 3 biological replicates. *P* values were determined by Student’s *t*-test. ** *P* < 0.01; *** *P* < 0.001. **d** Nuclear localisation of DII-Venus (middle panel) and mDII-Tomato (lower panel) from transiently expressed R2D2 in Col-0 petal protoplasts. The upper panel is the imaged protoplast in bright field. **e** Ratios of mDII-Tomato to DII-Venus transiently expressed in Col-O or *35S::KSK* petal protoplasts. Protoplasts from line OE1 were treated with 0 nM or 1000 nM IAA for 1–2 h before imaging. The increased ratio of mDII/DII shows reduced levels of DII-Venus in response to elevated auxin levels. *n* = 25 protoplasts were measured for each treatment in each of two independent experiments. *P* values were determined by Student’s *t*-test. ** *P* < 0.001. **f**. **f** and **g**. Reduced expression of the auxin-responsive genes *IAA1* (Panel F) and *IAA9* (Panel **g**) in developing petals of Col-0 and three transgenic lines overexpressing *KSK*. Expression levels are relative to *EF1ALPHA* gene expression. Data are given as means of +/−SE (*n* = 3 biological replicates. *P* values were determined by Student’s *t*-test. ** *P* < 0.01; *** *P* < 0.001

In addition to observing a significant increase in petal cell growth in the *35S::KSK* over-expressing lines, we also observed partial organ identity changes in ~ 10% of flowers from all eight *35S::KSK* transgenic plants (Additional File 6). Flowers with organ identity changes had an additional petal-like structure in the second whorl. This developed in the outer margin of the second whorl and displayed varying extents of stamenoid features such as a partial pollen sac (Fig. 6a) and stomata, a cell type not observed in Col-0 petals (Fig. 6b). Stamen numbers and development in the third whorl were normal in these flowers. The presence of stomata on petal epidermal surfaces has also been seen in *ant* mutants deficient in the transcription factor *AINTEGUMENTA* [23]. Using qRT-PCR, we assessed *ANT* expression in developing petals of the three *35S::KSK* over-expressing lines. A significant decrease in *ANT* expression was observed in petals overexpressing *KSK* (Fig. 6c). This suggests that *KSK* expression levels contribute to determining floral organ identity in a pathway involving *ANT*.

### Reduced auxin responses in lines over-expressing *KSK*

One common feature of *ARF8* and *FRL1* expression, which was suppressed by over-expression of *KSK*, is that the expression of both genes increases in response to auxin [20, 24, 25]. We therefore tested auxin responses in petals of wt Col-0 and *35S::KSK* over-expressing lines. The DII-n3Venus reporter protein is a fusion of the auxin-dependent DII degron to a nuclear-localised Venus reporter coding region. Auxin levels are detected by reduced Venus fluorescence relative to a mutant form, mDII-ntdTomato, which is not degraded in the presence of auxin [26]. This dual auxin reporter, called R2D2, is suitable for single cell assays as different transformation efficiencies can be accounted for by the relative fluorescence of nuclear-localised Venus and Tomato fluorescent proteins. Protoplasts were isolated from developing petals from Col-0 and a *35S::KSK* transgenic line over-expressing *KSK* and transfected with the R2D2 plasmid. After 16 h to allow for protein expression, protoplasts were treated with either 0 nM or 1000 nM IAA. Protoplasts were imaged between 1 and 2 h after IAA treatment. The nuclear localisation of both fluorophores is shown in Fig. 6d, while Fig. 6e shows relative fluorescence of mDII-Tomato/DII-Venus. The ratio of mDII-Tomato to DII-Venus was significantly increased in Col-0 protoplasts, demonstrating that transiently-expressed petal protoplasts respond to added auxin similarly to stable transgenic plants [26]. In contrast, in *35S::KSK* transgenic protoplasts, the ratio of Venus to Tomato fluorescence was not decreased to the same extent as Col-0. This indicated that auxin responses may be reduced in this transgenic line. This interpretation was tested by measuring the expression levels of two auxin- responsive genes (*IAA1* and *IAA9*) in petals of Col-0 and *KSK* over-expressing lines. Their expression was reduced (Figs. 6f, g), supporting the interpretation that auxin responses are decreased in *KSK* over-expressing lines.

## Discussion

Over 7000 *Arabidopsis thaliana* accessions have been systematically collected and over 1000 of these have been sequenced to access a wide range of genomic diversity [13]. We used genome-wide association analyses [27] on a relatively well-characterised set of accessions collected from Sweden [14] to identify a major source of sequence variation influencing petal size. Variation in the expression of a previously hypothetical gene encoding a plasma membrane protein, now called *KSK*, made a major contribution to variation in petal size in this set of accessions. Auxin responses as measured by the R2D2 reporter were reduced in *KSK* over-expression lines, suggesting that the KSK membrane protein may directly or indirectly influence auxin responses or levels in developing petals.

*KSK* was predicted to encode a transmembrane protein with 6 short helical domains spanning the membrane, 3 non-cytoplasmic domains and 4 cytoplasmic domains in an N-in conformation (Fig. 3b). A KSK-GFP fusion protein was localized to the plasma membrane (Fig. 3c). The KSK protein sequence is reasonably highly conserved among several groups of plants, and it has no close family members in Arabidopsis, with only very partial protein alignments to At1g31130 detected by reciprocal BLASTP searches. At1g31130 is a 321aa predicted 6TM protein located in Golgi, endosomes and the plasma membrane. The 6TM protein structure is predicted to be present in many Arabidopsis proteins with diverse functions, including aquaporins, voltage-gated ion superfamily transporters, and mitochondrial carrier proteins [28]. The MIND1 database of membrane protein interactions [29] identified an interaction between *KSK* and At1g07860, encoding a predicted Receptor-Like Cytoplasmic Kinase VII (RLCK VII) family member. Members of this family function in pathogen- triggered immunity and growth pathways [30] and include the relatively well characterised RLCK VII BIK1, which is membrane anchored via N-myristoylation [31]. In *KSK* over-expressing lines, auxin responses were reduced as detected by transient expression of the R2D2 auxin reporter gene (Fig. 6e). This may be due to reduced auxin responses, synthesis, or altered transport. The membrane localisation of KSK-GFP suggests a potential influence on auxin transport. However, the predicted 6TM transmembrane organisation of KSK in membranes is different from that of all known auxin uptake and efflux plasma membrane- and tonoplast-located auxin transporters [32].

Multiple promoter polymorphisms were identified between the small petal accession Col-O and the large petal accession Dju-1 (Fig. 3a and Additional File 5). In contrast, the protein coding regions of these two accessions had only two non-conserved amino acid changes (Fig. 3b); one was in a predicted non-cytoplasmic domain and the other in the predicted C-terminal non-cytoplasmic domain. While these may influence *KSK* function in different accessions, the phenocopying of the big petal phenotype of Dju-1 by over-expressing the Col-0 coding region indicated that promoter variation that increases expression most likely causes the large petal phenotype in the screened accessions. An interesting feature of the Dju-1 promoter region is the accumulation of expanded dA:dT-rich tracts of over 50 bp. Polymorphisms of this length are very common in Arabidopsis genome assemblies [33], and are over-represented in many eukaryotic genomes, where they may be generated by replication slippage [34]. dA:dT tracts in promoters have a well-established role in regulating gene expression by forming part of scaffold attachment regions (SARS) and by introducing curvature in DNA that influences transcription factor and nucleosome access.

*KSK* over-expression led to reduced expression of *ARF8* and *FRL1*, two genes that exert a specific negative effect on petal cell size (Figs. 5a, b, c). *FRL1* encodes a sterol methyltransferase that influences endoreduplication [35]. ARF8 is an auxin-responsive transcription factor that forms a transcription complex with the bHLH transcription factor BPEp [20]. BPEp also restricts cell expansion specifically in petals. BPEp is highly expressed during the later stages of petal development, while ARF8 is ubiquitously expressed, but more highly expressed during the later stages of petal development. The expression of both *FRL1* and *ARF8* was increased in response to auxin [24, 25, 36], and as auxin responses are reduced in *KSK* over-expressing lines (Fig. 6e), it is possible that *KSK* over-expression may reduce expression of these auxin-responsive negative regulators of petal cell size, leading to increased petal cell size and overall increases in petal area. The down-regulation of *ANT* expression in *KSK* over-expression lines (Fig. 4c) is consistent with reduced auxin responses, as auxin increases *ANT* gene expression [37]. *ANT* encodes an AP2/ERF transcription factor that influences several stages of floral development, including specification of floral organ identity. Petals in *KSK* over-expressing lines often exhibited a partial conversion to stamenoid features, and also had stomata, a cell type not normally found in petals (Figs. 6a, b). In *ant* mutants petal cell identity was also altered to form stomata [22, 23], supporting the conclusion that *KSK* modulates *ANT* expression, perhaps by altered auxin responsiveness, leading to partial homoeotic conversion of petals to stamenoid features. Such conversion is consistent with another function of *ANT* in excluding, together with *AP2*, *AGAMOUS (AG)* expression from the second whorl [23]. AG activates expression of *SPOROCYTELESS/NOZZLE (SPL/NZZ)*, a transcription factor that promotes microsporogenesis [38]. A lessening of this restriction of *AG* expression could therefore conceivably lead to stamenoid features developing on *KSK* over-expressing petals.

Floral morphology plays a central role in plant fitness, for example by attracting specific pollinators. In *Brassica napus* crops, reduced petal size is an important trait as it increases light penetration through dense canopies. In wild populations of *A. thaliana*, the adaptive significance, if any, of varying petal areas is not well understood. Although *Arabidopsis thaliana* is primarily self-pollinating, some populations exhibit elevated outcrossing, especially in species-rich rural environments [39]. Diverse insects visit Arabidopsis flowers and are potential pollinators [40], therefore it is reasonable to speculate that genetic variation in petal size may have an adaptive role in securing outcrossing in Swedish populations. The three lines selected with large petals came from different locations in southern Sweden (Additional File 1), all of which harbour accessions with varying sized petals. Natural variation in petal size in *A. thaliana* may therefore contribute to diversifying outcrossing opportunities by attracting different types of insect visitors.

## Methods

### Plant material and phenotyping

The 272 *Arabidopsis thaliana* accessions were obtained from Caroline Dean at the John Innes Centre. These are part of the 1001 Genomes Consortium study of *Arabidopsis thaliana* natural variation and are available from the Arabidopsis Biological Resources Centre under accession ID CS78942 [13]. All accessions, transgenic plants and T-DNA mutants were grown on soil in a growth chamber with 16/8 h day/night at 22 °C after 48 h stratification at 5 °C.

To quantify petal lengths, widths and areas, 10 petals were dissected from the first set of completely open flowers from 3 biological replicates per genotype to minimise any developmental differences. Petals were mounted on black cardboard and laminated to protect the petals. Petals were scanned at 200dpi resolution and petal length, maximum width and areas measured using Image J.

### GWAS

GWAS was carried out using the open-source GWAS software, GWAPP: https://gwas.gmi.oeaw.ac.at/ with the 250 K SNP dataset. Analyses were performed using the AMM function.

### DNA constructs

*p35S::3xHA-At4g16850* transgenic lines were created by cloning *At4g16850* cDNA into the pENTR TOPO-D vector (Thermofisher, UK) using the primers described in Additional File 7. The *At4g16850* CDS was transferred into the 35S PB7HA binary vector using LR Clonase mix II (Thermofisher, UK). The pAt4g16850::At4G16850-GFP transgenic line was generated by cloning the promoter and coding region from genomic DNA into the pENTR TOPO-D vector and LR clonase was then used to transfer the target sequence into the pEARLYGate 103 vector. All constructs were sequenced before use. The *p35S::3XHA-At4g16850* construct was transformed into *Agrobacterium tumefaciens* strain GV3101, and Arabidopsis *Col-0* plants were transformed using the floral dip method [41].

### Genotyping Arabidopsis T-DNA lines

Sequence indexed T-DNA insertion lines, obtained from The Nottingham Arabidopsis Stock Centre (NASC), were genotyped using gene-specific primers designed using the primer design tool http://signal.salk.edu/tdnaprimers.2.html: Primer sequences are in Additional File 7. Genotyping was carried out using TAKARA EX taq (Takara Bio, USA) according to the manufacturer’s instructions.

### cDNA synthesis, PCR and genome sequencing

RNA was extracted from developing petals dissected from developing floral buds or 12- day- old seedlings using the SPECTRUM Total Plant RNA kit (Sigma, UK). 1 μg of RNA was incubated with RQ1 RNase-Free DNase (Promega, USA) before cDNA synthesis. This used GoScript Reverse Transcription (Promega, USA) with OligoDT. cDNA samples were diluted 1:10 in water before use. Q-RT-PCR was performed using SYBR green mastermix (Thermofisher) and on a Lightcycler 480 (Roche, Switzerland). Additional File 7 describes the primers used for q-RT -[42]PCR. Primer efficiencies and relative expression calculations were performed according to . All q-RT-PCR assays were repeated in triplicate at least twice. All PCR reactions were carried out using Phusion High Fidelity DNA polymerase (New England BioLabs) according to manufacturer’s instructions. Capillary sequencing was carried out by GATC Biotech (Germany). For whole genome assembly of accessions Dju-1, T880 and TI070, high MW DNA was prepared using Qiagen columns and PCR-free indexed Illumina libraries prepared as described [43]. After QC approximately 50 m 150 bp paired-end reads were generated (Novagene, Hong Kong) for each library. Cleaned reads were assembled using ABySS v1.3.6 [44] with a *k*-mer size of 75. Genome assemblies were aligned with the genomic region of At4g16850 using MUSCLE v3.8.31 [45]. Assemblies of the three accessions are available at ENA (PREJB28030).

### Scanning Electron microscopy

Petals were dissected, fixed and critical point dried before SEM imaging. Chemical fixation was in 2.5% glutaraldehyde in 0.05 M sodium cacodylate, pH 7.4. Vacuum infiltration was carried out until the petals sank before leaving overnight in fixative at 4 °C. After rinsing in buffer twice and then water twice for 15 min each, petals were dehydrated through an ethanol series for 30 min each in 30, 50, 70, 90, 100, and 100% dry ethanol, then critical point dried using a Leica EM CPD300 system (Leica Microsystems Ltd., Milton Keynes, UK) according to the manufacturer’s instructions. Dried samples were mounted on the surface of an aluminium pin stub using double-sided adhesive carbon discs (Agar Scientific Ltd., Stansted, Essex). The stubs were then sputter coated with approximately 15 nm gold in a high-resolution sputter coater (Agar Scientific Ltd) and transferred to a Zeiss Supra 55 VP FEG scanning electron microscope (Zeiss SMT, Germany). The samples were viewed at 3 kV and digital TIFF files were stored.

### Transient expression in Arabidopsis protoplasts

Transient expression assays were carried out using protoplasts isolated from *Arabidopsis* Col-0 developing petals [46]. Protoplasts were transformed with 5μg plasmid DNA purified using the Qiagen Plasmid Maxi Kit (Qiagen). After an overnight incubation at 20 °C, transfected protoplasts were harvested and imaged using confocal microscopy. The R2D2 plasmid (Addgene 61,629 pGreenIIM RPS5A-mDII-ntdTomato/RPS5A-DII-n3Venus) was transfected into petal protoplasts isolated from Col-0 or *35S::KSK* plants. After overnight incubation, protoplasts were treated with 0 or 1000 nM IAA for 1–2 h before imaging. A Leica SP5 set up for photon counting at 12-bit resolution was used for imaging transfected protoplasts. Gain was set to 50% for Tomato fluorescence and to 10% for Venus fluorescence. Venus was excited at 514 nm and detected at 524-540 nm, and Tomato was excited at 561 nm and detected at 571-630 nm. ImageJ was used to calculate the mean gray value of fluorescence within nuclei.

## Supplementary information

**Additional file 1 **List of *Arabidopsis thaliana* accessions used in the GWAS analysis.

**Additional file 2.** Petal phenotype data.

**Additional file 3.** Representative petals from the Swedish accessions. (PPTX 3030 kb)

**Additional file 4.** Q-RT-PCR analysis of At4g16850 in seedlings of Col-0, Dju-1, T880 and T1070.

**Additional file 5 **Promoter alignments of the Dju-1 and Col-0 *KSK* genes.

**Additional file 6 **Organ counts in Col-0 and *35S::KSK* flowers.

**Additional file 7.** Primer sequences used in this study.

## Data Availability

Sequence reads of the *A. thaliana* accessions Dju-1, TBA_01 and TI-070 are available at ENA (PRJEB28030). Plant materials are available from the corresponding author.

## Abbreviations

AbySS: Assembly by Short Sequencing
FDR: False Discovery Rate
GWAS: Genome Wide Association Studies
LD: Linkage Disequilibrium
RIL: Recombinant Inbred Line
q-RT-PCR: Quantitative Reverse Transcription Polymerase Chain Reaction
QTL: Quantitative Trait Locus
SEM: Scanning Electron Microscopy
SNP: Single Nucleotide Polymorphism
