# Supplementary figures and images for: Variation in the expression of a transmembrane protein influences cell growth in Arabidopsis thaliana petals by altering auxin responses
Source: BMC Plant Biol. 2020 Oct 22;20:482. doi: 10.1186/s12870-020-02698-5 (PMC7584087; doi:10.1186/s12870-020-02698-5)

## Slide 1
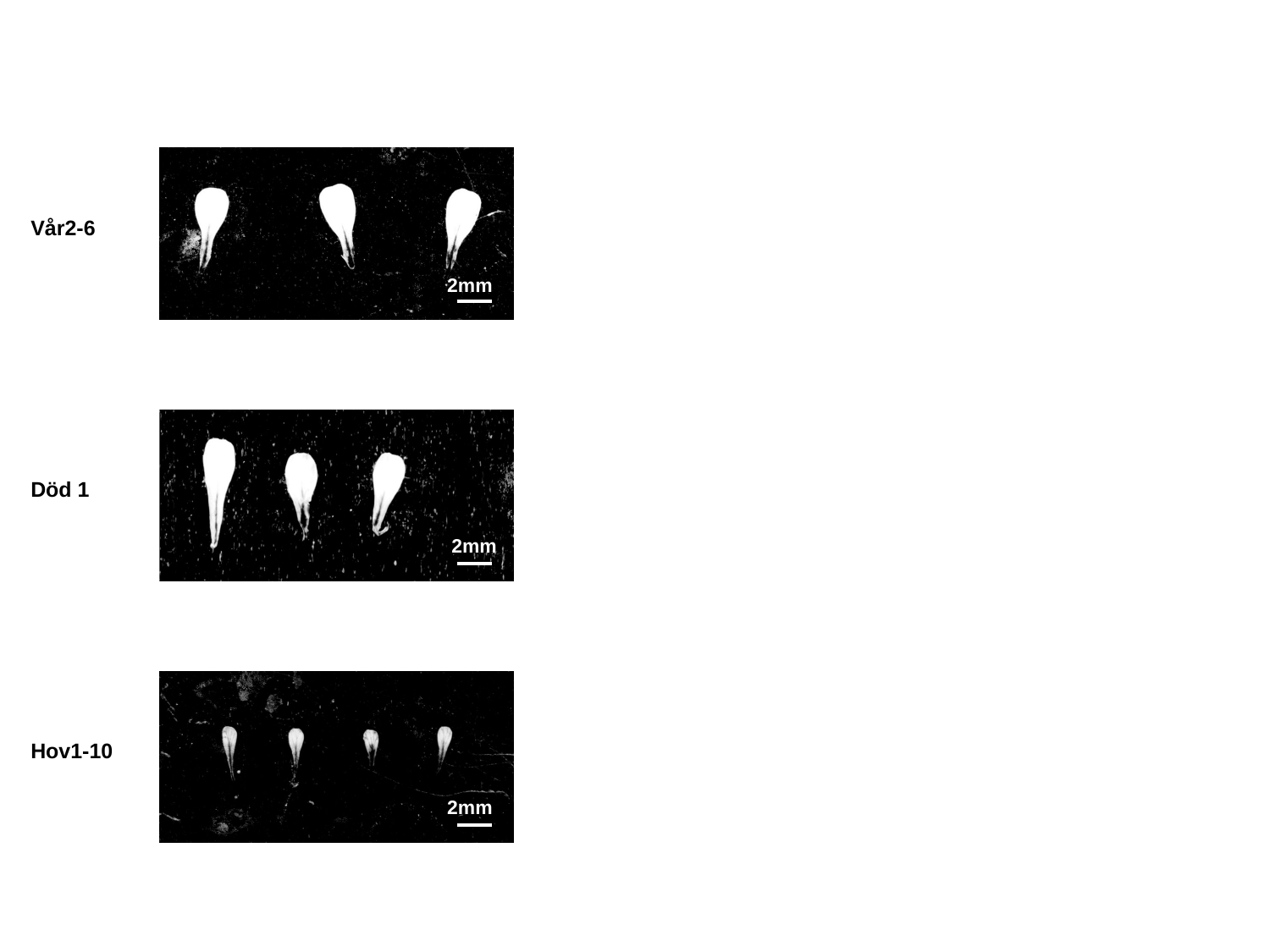

Vår2-6
2mm
Död 1
2mm
Hov1-10
2mm

Supplement: Supplementary file 3 — Additional file 3. Representative petals from the Swedish accessions. (PPTX 3030 kb) [file 12870_2020_2698_MOESM3_ESM.pptx]
