# Supplementary material for: Variation in the expression of a transmembrane protein influences cell growth in Arabidopsis thaliana petals by altering auxin responses
Source: BMC Plant Biol. 2020 Oct 22;20:482. doi: 10.1186/s12870-020-02698-5 (PMC7584087; doi:10.1186/s12870-020-02698-5)
Supplement: Supplementary file 4 — Additional file 4. Q-RT-PCR analysis of At4g16850 in seedlings of Col-0, Dju-1, T880 and T1070. [file 12870_2020_2698_MOESM4_ESM.docx]

**Additional File 4**. Q-RT-PCR analysis of At4g16850 in 12- day old seedlings. Col-0 was selected as a representative low-expressing *A. thaliana* accession.
