# Supplementary material for: Variation in the expression of a transmembrane protein influences cell growth in Arabidopsis thaliana petals by altering auxin responses
Source: BMC Plant Biol. 2020 Oct 22;20:482. doi: 10.1186/s12870-020-02698-5 (PMC7584087; doi:10.1186/s12870-020-02698-5)
Supplement: Supplementary file 5 — Additional file 5 Promoter alignments of the Dju-1 and Col-0 KSK genes. [file 12870_2020_2698_MOESM5_ESM.docx]

DJU1_KSK 1365 AAATTCCAGGTGTTGGAGATTGTTTTTGATTAAGCTATGGAACCATGGAC 1414

|||||||||||||||||||||||||||||||||.||||||||||||||||

TAIR10_KSK 1401 AAATTCCAGGTGTTGGAGATTGTTTTTGATTAAACTATGGAACCATGGAC 1450

DJU1_KSK 1415 TTGTCGACTCAGCCACCATCAACAACTGCAATACCATCCTCGAGAATTGC 1464

||||||||||||||||||||||||||||||||||||||||||||||||||

TAIR10_KSK 1451 TTGTCGACTCAGCCACCATCAACAACTGCAATACCATCCTCGAGAATTGC 1500

DJU1_KSK 1465 CGTAATAGCTCAGACACCACCACCACCAACAACAACAACAGTGTGGATCG 1514

|||||||.||||| .|||.|.|||||||||||||||||||||||.

TAIR10_KSK 1501 CGTAATACCTCAG------TCACTAACAACAACAACAACAGTGTGGATCA 1544

DJU1_KSK 1515 TCCCAGTGACTCAAACACCAACAACAATAACAGTGTGGATCATCCTAATG 1564

||||||||||||||||||||||||||||||||.||||||||||||.||||

TAIR10_KSK 1545 TCCCAGTGACTCAAACACCAACAACAATAACATTGTGGATCATCCGAATG 1594

DJU1_KSK 1565 ACATAAACAACAAGAACAATGTTGACAACAAGGACAATAACAGCAGAGAC 1614

|||||||.||||||||||||||||||||||||||||||||||||||||||

TAIR10_KSK 1595 ACATAAAAAACAAGAACAATGTTGACAACAAGGACAATAACAGCAGAGAC 1644

DJU1_KSK 1615 AAGTAATTAAGTAGGAAAAATCT-CGGTTTAGATGATACCG----ATCGG 1659

||||||||||.|||||||.| || ||||||||||||||||| |||||

TAIR10_KSK 1645 AAGTAATTAAATAGGAAACA-CTCCGGTTTAGATGATACCGATCTATCGG 1693

*VRN2* term.

DJU1_KSK 1660 ATTGTAACTTATTCTTCTTTCTTAAAAAAATTGTTTAGGAGCAAACGAA- 1708

||||||||||||||||||||||||||||||||||||||||||||||.||

TAIR10_KSK 1694 ATTGTAACTTATTCTTCTTTCTTAAAAAAATTGTTTAGGAGCAAACAAAG 1743

DJU1_KSK 1709 ATTTTAATTTGTTAGTGTGTATTCAACTGATTACATTTTTAGTT--GAAA 1756

||||| |||||||| |||||||||||||||||||||||||||| .|||

TAIR10_KSK 1744 ATTTT-ATTTGTTA--GTGTATTCAACTGATTACATTTTTAGTTAAAAAA 1790

DJU1_KSK 1757 ATGAATCCTGTTTAATTATTATTTTTTAATTAAAAACTGAAAAATTAAGA 1806

|||.||.||..|||| |||.||||.||||||||| |||||

TAIR10_KSK 1791 ATGGATTCTCCTTAA-----------TAACTAAAGACTGAAAAA-TAAGA 1828

DJU1_KSK 1807 AAAGTTTACTTAGTTTTTCTTTATGACTTGAGAAAAAGCT--------CC 1848

.||||||.||||.|||||||||.||||||||||||||||| ||

TAIR10_KSK 1829 TAAGTTTCCTTAATTTTTCTTTTTGACTTGAGAAAAAGCTCCTCTAGACC 1878

DJU1_KSK 1849 TCTCGTCAATAGGAGTTATATATAGATCAACTACATAACATAAATATATA 1898

|||.||.||||||||||||||||..|||||.||||||||||||

TAIR10_KSK 1879 TCTAGTAAATAGGAGTTATATATTAATCAAGTACATAACATAA------- 1921

DJU1_KSK 1899 TATATATATATATATATATATATATATATATATATTAAGTGCAAATAGAT 1948

|.|||||||||||||||||||||||||

TAIR10_KSK 1922 -----------------------AAATATATATATTAAGTGCAAATAGAT 1948

DJU1_KSK 1949 TGAAAACAAATCAAGAAATTAATTAAGACAGAGTGATTAAGCTTAAAACC 1998

||||||||||||||||||||||||||||||.|||||||||||||||||||

TAIR10_KSK 1949 TGAAAACAAATCAAGAAATTAATTAAGACACAGTGATTAAGCTTAAAACC 1998

*KSK* txn start site

DJU1_KSK 1999 CC 2000

||

TAIR10_KSK 1999 CC 2000

**Additional File 5.** Alignment (EMBOSS Needle) of *KSK* sequences upstream of the transcription start site in ecotypes Col-0 (smaller petals) and Dju-1 (larger petals).
