# Supplementary material for: Variation in the expression of a transmembrane protein influences cell growth in Arabidopsis thaliana petals by altering auxin responses
Source: BMC Plant Biol. 2020 Oct 22;20:482. doi: 10.1186/s12870-020-02698-5 (PMC7584087; doi:10.1186/s12870-020-02698-5)
Supplement: Supplementary file 6 — Additional file 6 Organ counts in Col-0 and 35S::KSK flowers. [file 12870_2020_2698_MOESM6_ESM.docx]

| **line** | **sepal** | **petal** | **stamenoid**  **petals** | **stamen** |
| --- | --- | --- | --- | --- |
| Col-0 | 4 | 4 |  | 6 |
| *35S::KSK 1* | 4 | 4 | 0.05 | 6 |
| *35S::KSK 2* | 4 | 4 | 0.10 | 6 |
| *35S::KSK 3* | 4 | 4 | 0.10 | 6 |

**Additional File 6.** Organ counts in flowers of Col-0 and *KSK* over-expressing lines.

20 early-stage flowers were measured and averages are shown.
